# Influence of Spinopelvic Alignment on the Clinical Outcomes Following Decompression Surgery for Lumbar Stenosis

**DOI:** 10.7759/cureus.46302

**Published:** 2023-10-01

**Authors:** Eyüp Varol

**Affiliations:** 1 Neurological Surgery, Umraniye Training and Research Hospital, Istanbul, TUR

**Keywords:** lumbar lordosis, pelvic tilt, sagittal balance, clinical outcomes, lumbar decompression surgery, sagittal spinopelvic alignment, lumbar spinal stenosis (lss)

## Abstract

Introduction

The role of sagittal spinopelvic alignment in lumbar spinal stenosis (LSS) patients and its potential influence on post-decompression surgery outcomes is a topic of growing interest. Lumbar spinal stenosis is a prevalent degenerative condition, and with an aging population, the frequency of surgical interventions for LSS has risen. While decompression surgery aims to relieve symptoms, the potential impact of preoperative spinopelvic alignment on postoperative results remains controversial. This study examined the correlation between sagittal spinopelvic parameters and clinical outcomes in LSS patients undergoing decompression surgery.

Methods

This study included 100 patients with LSS who underwent decompression surgery between 2021 and 2023 and 100 healthy individuals as a control group. The LSS group comprised 50 men and 50 women, with a mean age of 55.8±12.41 years, while the control group consisted of 50 men and 50 women, with a mean age of 55.17±13.39 years. Sagittal spinopelvic alignment parameters, including pelvic tilt (PT), pelvic incidence-lumbar lordosis mismatch, and sagittal vertical axis, were assessed preoperatively. Postoperative clinical outcomes were evaluated using the visual analog scale (VAS) and Oswestry disability index (ODI) scores.

Results

In the cohort of 200 participants, 100 were diagnosed with lumbar spinal stenosis (LSS), and 100 were healthy controls. Both groups had an equal gender distribution (50 males and 50 females). The mean age was 55.8 (±12.4) years for the LSS group and 55.2 (±13.4) years for the control group. Among the analyzed radiographic parameters, only lumbar lordosis (LL) levels showed a significant difference between groups, notably lower in the LSS group (p=0.020). Preoperative VAS scores in LSS patients averaged 7.58±1.32, which postoperatively dropped to 2.22±1.95 (p<0.001). Similarly, ODI (%) declined from a preoperative average of 55.76±11.65 to 18.62±18.17 postoperatively (p<0.001). Patients with postoperative ODI levels exceeding 20% had higher preoperative scores and significantly altered radiographic measurements. The receiver operating characteristic (ROC) analysis indicated PT as the most predictive radiographic parameter, with an area under the curve (AUC) of 0.945. Multivariate logistic regression pinpointed PT and LL as key predictors associated with increased risks for postoperative Oswestry disability levels exceeding 20%.

Conclusion

Our study suggests that sagittal spinopelvic alignment plays an important role in the development and progression of LSS. Addressing sagittal alignment may be crucial for achieving optimal clinical outcomes after decompression surgery. Further research is needed to elucidate the mechanisms underlying the relationship between sagittal alignment and LSS.

## Introduction

There is thought to be a relationship between sagittal spinopelvic alignment and clinical outcomes in patients with lumbar spinal stenosis (LSS) who undergo decompression surgery [1]. Lumbar spinal stenosis is a degenerative spine disorder characterized by the narrowing of the spinal canal, which often results in nerve compression, pain, and functional impairment. As the population ages, the prevalence of LSS is increasing, and it has become a leading cause of spinal surgery in older adults [2]. The main goal of surgical intervention for LSS is to alleviate the symptoms of nerve compression by removing the structures causing the narrowing, typically through a procedure known as laminectomy or decompression surgery [1-3].

Sagittal spinopelvic alignment's importance in spine health and function has been increasingly recognized in recent years. Spinopelvic alignment refers to the orientation of the pelvis and spine in the sagittal plane and is characterized by parameters such as pelvic tilt (PT), sacral slope (SS), and lumbar lordosis (LL). Abnormal spinopelvic alignment has been associated with various spinal pathologies, including LSS, and has been shown to affect spinal biomechanics, posture, and stability [3].

Numerous studies have explored the potential impact of sagittal spinopelvic alignment on the clinical outcomes of spinal surgeries, including decompression surgery for LSS [4,5]. Understanding the relationship between spinopelvic alignment and surgical outcomes is paramount for optimizing surgical planning and enhancing postoperative outcomes. However, the evidence on this topic has been inconsistent, with some studies reporting a correlation between sagittal alignment and postoperative outcomes, while others have found no such association [6].

Given the increasing recognition of sagittal spinopelvic alignment's role in spine health, its potential influence on clinical outcomes after decompression surgery for LSS is of significant interest. With the literature presenting conflicting evidence, gaining clarity on the relationship between spinopelvic alignment and postoperative outcomes becomes pivotal. A clear understanding of this area is academically enriching and clinically essential. It holds implications for tailoring patient-specific surgical approaches, guiding postoperative care, and improving patient outcomes. Our study explicitly addresses this knowledge gap, aiming to discern the nuances of the impact of spinopelvic parameters, such as PT, SS, and LL, on postoperative results in LSS patients. By comparing these parameters and their outcomes in LSS patients against a healthy control group, we hope to shed light on the intricacies of spinopelvic alignment in the context of LSS management and further the discourse on evidence-based surgical practices.

## Materials and methods

Study design and participants

This retrospective cohort study was conducted on 100 patients diagnosed with lumbar spinal stenosis who underwent decompression surgery between 2021 and 2023. The study included 50 female and 50 male patients. A control group was also established, consisting of 100 age-matched and sex-matched healthy individuals without spinal pathologies. Patients and controls were selected from the same institution's outpatient clinics. The inclusion and exclusion criteria for the groups are stated in Table 1.

**Table 1:** Inclusion and exclusion criteria for LSS and control groups LSS: lumbar spinal stenosis

Inclusion criteria	Exclusion criteria
For the LSS group	
Age ≥ 18 years	History of other spinal surgeries (e.g., fusion, discectomy) in the LSS group
Clinical diagnosis of LSS confirmed by MRI or CT scan	Presence of other spinal pathologies, such as disc herniation, spondylolisthesis, or scoliosis, in the LSS group
Underwent decompression surgery for LSS between 2021 and 2023	Previous spinal trauma or deformity
Complete clinical and radiological data are available.	Rheumatoid arthritis, ankylosing spondylitis, or other systemic inflammatory conditions affecting the spine
	Only complete medical records or radiological data
	History of neuromuscular disorders affecting gait or posture
	Inability to provide informed consent or follow the study protocol
For the control group	
Age ≥ 18 years	Age <18 years
No history of spinal pathology or surgery	History of spinal pathology or surgery
Complete clinical and radiological data are available.	Lack of clinical and radiological data

Data collection

The medical records of eligible patients were reviewed to extract relevant demographic and clinical data, including age, sex, body mass index (BMI), preoperative symptoms, surgical details, and postoperative outcomes. Radiological imaging, including preoperative and postoperative MRI or CT scans, was assessed to confirm the diagnosis of LSS and evaluate the extent of decompression achieved. Spinopelvic parameters, including PT, SS, LL, and PI, were measured using standing lateral radiographs of the whole spine and pelvis. This tool facilitated a standardized measurement process and ensured that the extracted spinopelvic parameters were consistent and accurate. For these measurements, the RadiAnt DICOM Viewer software (Medixant, Poznan, Poland) was employed.

Statistical analysis

In this study, IBM Statistical Package for the Social Sciences (SPSS) version 26 (IBM Corp., Armonk, NY, USA) and R software V4.3.1 (R Core Team, Rstudio Team, 2020, Vienna, Austria) were used for statistical analysis. The normality of scores obtained from each continuous variable was examined through descriptive, graphical, and statistical methods. The Kolmogorov-Smirnov test was employed to test the normality of scores obtained from a constant variable with the aim of statistical evaluation. Categorical variables were presented as frequencies (n, %), while continuous variables were presented as mean ± standard deviation, median, and interquartile range. Comparisons between two groups for categorical and continuous variables were conducted using the chi-square and independent-sample t-tests, respectively. The paired sample t-test was employed to test the difference between two continuous variables in repeated measurements. Multivariable logistic regression modeling was utilized to measure the effect of independent variables on the dependent variable (ODI level). The area under the curve (AUC) values and diagnostic performance of radiographic parameters used to predict the presence of disability (>20%) were calculated using receiver operating characteristic (ROC) analysis. The strength of the relationship between dependent and independent variables and the goodness of fit of the established model was assessed using the Hosmer-Lemeshow test and the Nagelkerke R square statistic. Results were considered significant at p < 0.05 with a confidence interval of 95%.

Ethical considerations

The study was conducted in accordance with the Declaration of Helsinki and was approved by the ethics committee of the University of Health Sciences Ümraniye Training and Research Hospital, Neurosurgery Clinic, Istanbul, Turkey, where the study was conducted (approval number: B.10.1.TKH.4.34.H.GP.0.01/981). All participants gave written informed consent to participate in the study and to have their medical records reviewed for research purposes. The data were de-identified to ensure confidentiality.

In this study, we aimed to explore the impact of sagittal spinopelvic alignment on clinical outcomes following decompression surgery for LSS by comparing spinopelvic parameters and postoperative results between LSS patients and a control group of healthy individuals without spinal pathologies.

## Results

Demographic and radiographic parameters

This study included a total of 200 participants, comprising 100 patients diagnosed with LSS [1] and 100 healthy individuals in the control group. The mean age of the LSS group was 55.8 (±Sd:12.4) years, while the control group had a mean age of 55.2 (±Sd:13.4) years (p>0.05). Both study groups consisted of 50 males and 50 females. When analyzing the distribution of BMI and radiographic parameters (PT, LL, SS, and pelvic incidence (PI)) between groups, a significant difference was observed only in LL levels (p<0.05). The LL levels were statistically significantly lower in the LSS group (p=0.020) (Table 2).

**Table 2:** Comparison of demographic and radiographic parameters according to groups *P<0.05; #mean±standard deviation; PT: pelvic tilt; LL: lumbar lordosis; SS: sacral slope; PI: pelvic incidence; BMI: body mass index; a: independent sample t-test; b: Chi-Square test; LSS: lumbar spine stenosis

Variables(N=200)	LSS (n=100)	Control (n=100)	P-value^a^
Age, year^#^	55.8±12.41	55.17±13.39	0.730^a^
Male/Female, n	50/50	50/50	1^b^
BMI^#^	27.69±2.41	27.25±2.98	0.254^a^
PT^#^	13.29±4.39	14.25±2.88	0.068^a^
LL^#^	46.23±12.80	50.10±10.32	0.020^a^*
SS^#^	40.65±7.72	40.84±9.03	0.873^a^
PI^#^	53.93±9.54	55.09±9.37	0.389^a^

The visual analog scale (VAS) and Oswestry disability index (ODI)(%) levels and postoperative changes

The mean preoperative VAS score for LSS patients was 7.58±1.32, which significantly decreased to 2.22±1.95 in the postoperative period (p<0.001). Similarly, the ODI(%) level decreased considerably from 55.76±11.65 preoperatively to 18.62±18.17 postoperatively (p<0.001) (Table 3).

**Table 3:** VAS and ODI(%) change for the postoperative and postoperative period *P<0.05 for paired samples t-test; IQR(P25-P75): interquartile range; CI: confidence interval; ODI: Oswestry disability index; VAS: visual analog scale; SD: standard deviation; CI: confidence interval

		Mean±SD	Median (IQR)
VAS	Preoperative	7.58±1.32	8(7-9)
	Postoperative	2.22±1.95	2(1-2)
	Mean diff.(95%CI)	-5.36[-5.67;-5,05]	-6[(-6)-(-5)]
	P-value	<0.001*	
ODI(%)	Preoperative	55.76±11.65	54(45-65)
	Postoperative	18.62±18.17	11(8-17.8)
	Mean diff.(95%CI)	-37.14[-40.31;-33,97]	-38.5[(-47.8)-(-29.3)]
	P-value	<0.001*	

Variables associated with postoperative ODI(%) level

The results of the univariate analysis indicated that 22 patients had ODI levels exceeding 20% in the postoperative period. Patients with ODI levels >20% had significantly higher preoperative VAS (p<0.001) and ODI (p=0.008) scores. Furthermore, patients with ODI levels >20% had statistically significantly lower radiographic parameter (PT, LL, SS, and PI) measurements compared to those with ODI levels ≤20% (p<0.01 and p<0.001) (Table 4).

**Table 4:** Variables associated with postoperative ODI(%) score (univariate analysis results) *P<0.05; #mean±standard deviation; PT: pelvic tilt; LL: lumbar lordosis; SS: sacral slope; PI: pelvic incidence; BMI: body mass index; a: independent sample t-test; b: chi-square test; ODI: Oswestry disability index; VAS: visual analog scale

		Postoperative ODI		
Variables	All, n	≤20% (n=78)	>20% (n=22)	P-value
Age, year#	100	55.92±12.39	55.36±12.73	0.853^a^
Male/Female, n(%)	50/50	39(78%)/11(22%)	39(78%)/11(22%)	1^b^
Preoperative VAS^#^	100	7.32±1.26	8.50±1.10	<0.001^a^*
Preoperative ODI(%)^#^	100	54.13±10.52	61.55±13.75	0.008*
BMI^#^	100	27.72±2.57	27.58±1.77	0.813^a^
PT^#^	100	14.96±2.93	7.34±3.46	<0.001^a^*
LL^#^	100	50.31±11.10	31.76±6.25	<0.001^a^*
SS^#^	100	41.72±7.89	36.84±5.78	0.008^a^*
PI^#^	100	56.69±8.45	44.18±6.30	<0.001^a^*

Results of ROC analysis 

**Figure 1:**
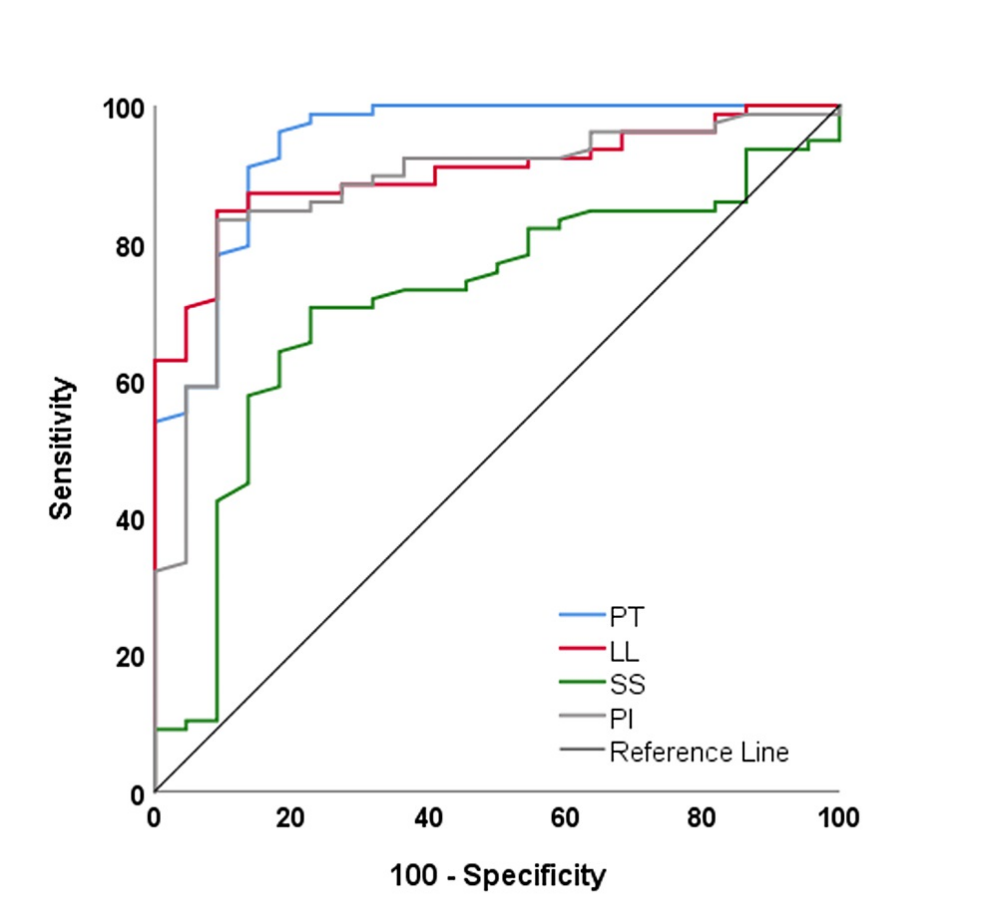
ROC curves for >20% disability ROC: receiver operating characteristic; PT: pelvic tilt; LL: lumbar lorosis; SS: sacral slope; PI: pelvic incidence

The ROC analysis results for predicting Oswestry disability levels >20% are detailed in Table 5 and Figure 1.

**Table 5:** Threshold value and diagnostic performance of radiographic parameters to predict the presence of >20% disability *p<0.05; CI: confidence interval; AUC: area under the curve; PT: pelvic tilt; LL: lumbar lordosis; SS: sacral slope; PI: pelvic incidence

	AUC (95% CI)	Cut off	P-value	Sensitivity (%)	Specificity (%)	Accuracy(%)
PT	0.945(0.888-1)	<11	<0.001*	81.8	96.2	93
LL	0.902(0.842-0.962)	<39.6	<0.001*	90.9	84.6	86
SS	0.714(0.596-0.831)	<38.9	0.002*	77.3	70.5	72
PI	0.882(0.805-0.956)	<50.1	<0.001*	90.9	83.3	85

The highest AUC value was obtained for PT (0.945), while the lowest was observed for SS (0.714). The threshold value for predicting >20% Oswestry disability was determined as 11 for PT (sensitivity=81.8%, specificity=96.2%, and accuracy=93%), 39.6 for LL (sensitivity=90.1%, specificity=84.6%, and accuracy=86%), 38.9 for SS (sensitivity=77.3%, specificity=70.5%, and accuracy=72%), and 50.1 for PI (sensitivity=90.9%, specificity=83.3%, and accuracy=85%).

Multivariate logistic regression model analysis results

**Figure 2:**
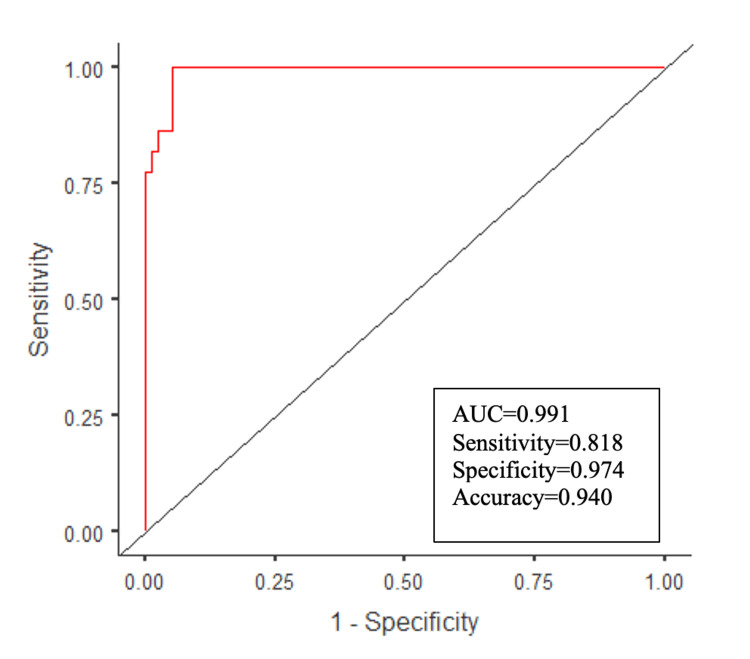
Multivariate logistic regression model ROC curve AUC: area under the curve; ROC: receiver operating characteristic

A multivariate logistic regression model was constructed, including variables (preoperative VAS and ODI levels, PT, LL, and PI radiographic parameters) that were statistically significantly associated with Oswestry disability levels >20% according to the univariate analysis. The SS radiographic parameter was excluded from the model due to its low AUC value. In this analysis, two independent factors associated with >20% Oswestry disability levels were identified. The model determination coefficient R2 (Nagelkerke) was found to be 0.87, indicating that the independent variables could explain 87% of the variance in the dependent variable. According to the Hosmer-Lemeshow test, the model fit the data well (χ2=2.706; p=0.951). According to ROC analysis, the AUC value of the regression model was 0.99, with an accuracy rate of 94%, sensitivity of 82%, and specificity of 97%. According to the multiple logistic regression model, increasing levels of PT (β=-0.887, odds ratio (OR)=0.412, p=0.003) and LL (β=-0.197, OR=0.821, p=0.002) radiographic parameters were associated with an increased risk of >20% Oswestry disability (Table 6 and Figure 2).

**Table 6:** Multivariate logistic regression model analysis results of radiographic parameters to predict the presence of >20% disability *P<0.01; multivariate logistic regression (backward stepwise model)' R2 (Nagelkerke)=0.874; model χ2=84.250, p<0.001; Hosmer and Lemeshow test p-value=0.951; Dependent variable=ODI(1=>20%, 0=≤20%); SE: standard error; OR: odds ratio; PT: pelvic tilt; LL: lumbar lordosis

		95% Confidence Interval						95% Confidence Interval	
Predictor	β	Lower	Upper	SE	Z	P-value	OR	Lower	Upper
Intercept^(Step4)^	16.731	7.014	26.448	4.958	3.37		1.85e+7	1111.710	3.06e+11
PT	-0.887	-1.475	-0.298	0.300	-2.95	0.003	0.412	0.229	0.743
LL	-0.197	-0.325	-0.070	0.065	-3.03	0.002	0.821	0.723	0.933

## Discussion

This retrospective cohort study investigated the impact of sagittal spinopelvic alignment on clinical outcomes following decompression surgery for LSS. We compared the spinopelvic parameters and postoperative results of 100 patients with LSS against a control group of 100 healthy individuals without spinal pathologies.

Our results demonstrated significant differences in spinopelvic parameters between the LSS and control groups, particularly in PT, SS, and LL. These findings align with previous research showing that alterations in spinopelvic alignment can contribute to the development and progression of spinal degenerative diseases, including LSS [7,8]. In our LSS group, an increase in PT and a decrease in SS and LL were observed compared to the control group. These alterations suggest a compensation mechanism that individuals with LSS adopt to reduce the pressure on the neural elements and alleviate their symptoms [8,9].

Postoperative outcomes in the LSS group significantly improved pain, function, and quality of life scores. The positive effect of decompression surgery on these outcomes has been well documented in the literature [1,10]. Our study revealed that better sagittal spinopelvic alignment correlated with more favorable postoperative outcomes. Specifically, patients with higher PT and ST and more pronounced LL had better postoperative outcomes. These results are consistent with prior studies that reported a strong association between sagittal spinopelvic alignment and clinical outcomes in LSS patients [11,12]. Maintaining an optimal spinopelvic alignment is crucial for distributing the mechanical load across the spine, which may contribute to better surgical outcomes [13,14].

Our findings have several clinical implications. First, they highlight the importance of assessing sagittal spinopelvic alignment in LSS patients undergoing decompression surgery, as it may influence the surgical outcome. Secondly, the results suggest that corrective measures to improve the sagittal alignment, such as physiotherapy or specific surgical techniques, could be considered part of managing LSS [15,16]. However, further research is needed to establish the optimal approach to addressing sagittal alignment in LSS patients and its potential impact on postoperative outcomes.

In conclusion, our discussion underscores the significant observation that preoperative LL angles in patients with LSS are significantly lower than in individuals with normal spinal alignment. This disparity in LL angles is closely associated with higher VAS and ODI scores in LSS patients. Moreover, in the LSS group, higher preoperative LL angles were associated with significant improvement in postoperative VAS and ODI scores.

Additionally, in light of the statistical data, it becomes evident that by examining the spinopelvic parameters, including PT, PI, LL, and SS, one can reasonably predict whether a patient's ODI score will exceed 20%. However, it is essential to acknowledge that further investigation involving a broader patient population must establish these values conclusively.

These findings hold considerable clinical significance, as they suggest that by assessing these spinopelvic parameters, clinicians can anticipate the trajectory of patient mobility, pain levels, and the extent of change in ODI and VAS scores following surgery. This predictive insight could be pivotal in preoperative planning and postoperative management, ultimately leading to more effective and personalized care for LSS patients.

There are several limitations to our study. First, the study's retrospective nature may have introduced selection and information bias. Second, more than the current sample size may be required to detect small effect sizes or conduct subgroup analyses. Third, the study did not consider other potential confounding factors, such as comorbidities or preoperative physical activity levels, which may have influenced the outcomes. Lastly, the follow-up period was limited, and long-term outcomes were not evaluated. Further prospective studies with larger sample sizes and more extended follow-up periods are needed to confirm these findings and explore the potential benefits of corrective measures to optimize sagittal alignment in LSS patients.

## Conclusions

Our retrospective cohort analysis delved into the intricate relationship between sagittal spinopelvic alignment and postoperative outcomes after decompression surgery in LSS patients. Distinct disparities in spinopelvic parameters emerged when comparing the LSS cohort to the control group. This observation underscores the potential significance of sagittal spinopelvic alignment in both the onset and advancement of LSS. Furthermore, the LSS patient group exhibited marked enhancements in postoperative pain levels, functional ability, and overall quality of life. Notably, superior postoperative outcomes were tightly interwoven with optimal sagittal spinopelvic alignment. Our data thus emphasize the imperative of considering sagittal alignment while planning surgical interventions for LSS, paving the way for maximized surgical benefits.
